# Autoimmune Mechanisms of Interferon Hypersensitivity and Neurodegenerative Diseases: Down Syndrome

**DOI:** 10.1155/2020/6876920

**Published:** 2020-06-01

**Authors:** Ashraya Jagadeesh, Leonard E. Maroun, Lisa M. Van Es, Richard M. Millis

**Affiliations:** ^1^Department of Microbiology and Immunology, American University of Antigua College of Medicine, St. John's, Antigua and Barbuda; ^2^Linda Crnic Institute for Down Syndrome, University of Colorado Hospital, Aurora, CO 80045, USA; ^3^Department of Pathophysiology, American University of Antigua College of Medicine, St. John's, Antigua and Barbuda

## Abstract

Down syndrome (DS), also known as trisomy 21 (T21), is associated with interferon (IFN) hypersensitivity, as well as predilections for Alzheimer's dementia (AD) and various autoimmune diseases. IFN-*α* and IFN-*γ* receptors are encoded on chromosome 21 (Ch21). It remains unclear how other Ch21 genes contribute to the neuropathological features of DS/T21. This study tests the hypothesis that identifying IFN-stimulated response element (ISRE) control sites on Ch21 will mark novel candidate genes for DS/T21-related IFN hypersensitivity and neuropathology not previously reported to be associated with IFN functions. We performed whole chromosome searches of online databases. The general ISRE consensus and gamma interferon activation consensus sequences (GAS) were used for identifying IFN-stimulated response elements. Candidate genes were defined as those possessing two or more ISRE and/or GAS control sites within and/or upstream of the transcription start site. A literature search of gene functions was used to select the candidate genes most likely to explain neuropathology associated with IFN hypersensitivity. *DOPEY2*, *TMEM50B*, *PCBP3, RCAN1*, and *SIM2* were found to meet the aforementioned gene search and functional criteria. These findings suggest that *DOPEY2*, *TMEM50B*, *PCBP3, RCAN1*, and *SIM2* are genes which may be dysregulated in DS/T21 and may therefore serve as novel targets for treatments aimed at ameliorating the neuropathological features of DS/T21. Future studies should determine whether these genes are dysregulated in patients with DS, DS-related AD, and autoimmune diseases.

## 1. Introduction

Trisomy 21 (T21) causes a variety of phenotypes known as Down syndrome (DS). DS is a condition characterized by intellectual disability associated with early neurodegeneration, Alzheimer's dementia (AD), interferon (IFN) hypersensitivity, and predilections for autoimmune diseases [1]. Autoimmune thyroid disease, celiac disease, type1 diabetes mellitus (DM1), and various autoimmune and autoinflammatory skin conditions are commonly found in DS/T21 patients [1]. IFN levels and IFN-inducible genes (IFN signature) appear to correlate with the pathological features of such autoimmune diseases [2].

IFNs are cytokine regulators of immune responsiveness, possessing antiviral activity primarily, but also having an activity that protects against a wide variety of neoplastic and inflammatory conditions, including autoimmune diseases [3]. IFNs are classified as type1 (alpha, beta, tau, and omega) or type2 (gamma) [4]. Based on the current knowledge, the only type1 IFNs that are significant for diseases in humans are IFN-*α*, IFN-*β*, and IFN-*γ*. Whereas the genes that code for type1 IFN-*α* are clustered on chromosome 9 (Ch9), the genes for type2 IFN-*γ* are found on Ch19 [5]. IFN responsiveness involves production of type1 and type2 IFNs. Type1 IFNs activate cells by dimerizing its specific cell surface receptors, IFN-*α*/*β* (IFNARs), made up of two polypeptide subunits named IFNAR1 and IFNAR2 that are expressed on all nucleated cells [3]. There are 13 subtypes of type1 IFN-*α*, each with somewhat different, but overlapping, functions for orchestrating an immune response in humans. All the genes encoding the 13 IFN-*α* subtypes reside on Ch9 [6]. There is only one IFN-*β* in humans which appears to be protective against the autoimmunity associated with multiple sclerosis. There is also one type2 IFN known as IFN-*γ*, which, similar to IFN-*α*, is recognized by binding and dimerizing its two specific receptor subunits, IFNGR1 and IFNGR2. One of the more interesting aspects of IFN-*γ* activity is its ability to inhibit the activity of T helper-2 (Th2) in favor of activating T helper-1 (Th1) lymphocytes, thereby inducing direct antiviral neutralizing and cytotoxic effects such as those implicated in autoimmune disease-related attack of myelin in multiple sclerosis [7]. IFN dysregulation also appears to be a factor in the development of inflammation in arterial plaques associated with atherosclerosis and in uterine decidua associated with spontaneous abortions, in persons affected by systemic lupus erythematosus (SLE) with antiphospholipid syndrome [8]. Type1 IFNs are also shown to play an important role in various autoimmune diseases [9]. Elevated serum levels of IFN-*α* are reported in patients diagnosed with SLE, whereas increased expression of IFN-*γ* is found in a mouse model of SLE [10, 11].

INFs exert their effects by interaction with the aforementioned cell surface receptors via activation of Janus kinase/signal transducer and activator of transcription (JAK/STAT) intracellular signal transduction proteins which, in turn, activates various transcription factors [12]. Transcription of IFN-stimulated genes involves the presence of a DNA element which is part of the IFN-response enhancer usually found in the 5' upstream regions of the genes [13]. This element, termed an IFN-*α* stimulated response element (ISRE), is composed of direct repeats of the sequence TTTC spaced by one or two nucleotides [14]. A common consensus for this sequence is AGTTTCNNTTTC [15]. The site termed the IFN-*γ* activated site (GAS) consists of a palindromic repeat of the sequence TTC spaced by three nucleotides; the consensus sequence is TTCCNNGAA [14]. All IFN types appear to mediate the binding of regulatory factors to ISRE sequences [14]. Both type1 and type2 IFNs can influence binding to GAS elements. There appears to be sequence heterogeneity among naturally occurring ISRE sites with varying affinity for different binding proteins [14]. Activated transcription factors, in turn, bind to upstream regulatory regions of IFN-inducible genes [16]. All of the type1 IFNs share the same receptor complex [4], whereas type2 IFN-*γ* has a distinct receptor. The IFN-*γ* receptor has been characterized and cloned. A gene encoding the ligand binding component of the receptor has been localized to Ch6, whereas a second component of the receptor has been localized to Ch21; both components of the receptor are required for its function [4, 17]. The presence of Ch21 confers sensitivity to type1 IFN [4]. This finding suggests that the IFN-*α* hypersensitivity associated with DS/T21 results directly from a trisomy-related increase in the number of IFN-*α* receptors.

The aforementioned results corroborate the finding that increased expression of IFN-stimulated genes is found in CD8+ T cells overproducing IFN-*γ* and TNF-*α* cytokine linked to autoimmunity [1]. IFN-stimulated ISRE and GAS control sites on Ch21 may, therefore, serve as markers for genes which have not been previously implicated in IFN functions. The present study is designed to test the hypothesis that IFN-stimulated ISRE and GAS control sites on Ch21 are markers for novel candidate genes that are dysregulated in DS/T21-related IFN hypersensitivity, neurodegeneration, and predilections for various autoimmune diseases. Identifying such genes may provide new targets for DS/T21 treatments.

## 2. Methods

We performed whole chromosome searches online using the NIH GenBank database of all publicly available DNA sequences. ISRE and GAS consensus sequences were used for identifying IFN-stimulated response elements in or near Ch21. Candidate genes were defined as those possessing two or more ISRE and/or GAS control sites within and/or upstream of the transcription start site (TSS). The entire chromosome 21 was searched for the consensus (AGTTTCNNTTTCNC) sequence. Genes that had two or more of this 14 bp sequence in or just upstream of a gene were chosen for further analysis. The entire Ch21 was then searched for the second consensus (AGTTTCNNTTTCNCT) sequence and genes that had two or more of this sequence were considered for further analysis. A functional ISRE site could be located quite far (>100,000 bp) from the transcription start site. The whole chromosome search using the ISRE consensus sequence provided a short list from the estimated 300–352 genes on Ch21 that met our inclusion criterion of containing at least two ISRE consensus sites within the gene and/or upstream of its TSS. A second set of genes that are considered candidate genes in DS/T21 neuropathology was assembled by doing a literature review and together this made up the final list of genes for a detailed analysis.

Each gene was screened with 22 different known ISRE sequences and nine known GAS sequences. This involved a double search: the first one was done using the first 10 bases of the ISRE and the second search involved the last 10 bases. The search was considered positive if a sequence containing 10 contiguous bases was discovered in the vicinity of a TSS or within a gene. Upon finding a 10-base sequence, it was further examined to see if the homology with the ISRE extended beyond 10 bases. This process was repeated for each gene using each known individual ISRE sequence. A second list of GAS sequence known to function in IFN-*γ* regulation was assembled from the literature. The GAS sequences are relatively shorter than the ISRE. In the case of the shorter GAS sequence analysis, the search was done using nine bases (either first nine or last nine). Each site was examined for extended homology by one or more bases. In the final analysis, only genes exhibiting complete contiguous homology with a known GAS sequence were included. When ISRE/GAS sequences were found within the gene, some of them had these sequences upstream to the TSS while in others they were found either inside of the gene or downstream toward the end of the gene. For the sequences found upstream of the TSS, it was important that these sequences were found no farther than 10 to 15,000 base pairs from the TSS. For the ISRE to be considered significant, sequences found had to show an 11-base contiguous homology for a 13-base sequence used in the search. In the case of a 15-base search sequence, the gene had to have an ISRE with extended contiguous homology of up to 13 contiguous bases. The above criteria also required the mandatory presence of the conserved repeat TTTCNNTTTC or TTTCNTTTC sequences within the ISRE. Several genes such as *INDO, MX1, CXCL10, PRKR*, and *ADAR1* have been previously shown to be IFN-regulated. These genes served as positive controls. Genes that have been previously shown to be downregulated by IFN such as *PTX3, COX17, TRAF6, KIAA0217*, and *BCR* served as negative controls.

## 3. Results

The aims of this study are to identify novel IFN-regulated genes on Ch21 which are most likely to be involved in the neuropathology and autoimmune dysfunction associated with DS/T21. The entire chromosome 21 was scanned using the ISRE consensus sequences to develop a short list of candidate genes for a more detailed analysis using known ISRE and GAS sequences (Tables 1 and 2).

Both the receptors for type1 IFN as well as one of the receptors for type2 IFN are located on chromosome 21. ISRE and GAS sequences reported here for the group of genes by the analysis to be IFN-regulated are in fact also predicted to be upregulated. This conclusion is based on the observation that the upregulated genes showed a similar pattern of distribution as the predicted group (PR). There was a notable lack of such distribution within genes that are known to be downregulated by IFN.

Genes that had the highest positive predictive value had to have met the strict criteria of (1) having multiple ISRE and at least one complete GAS sequence, (2) being involved in brain, immunologic, or immunity-related developmental function, and (3) not previously demonstrating to be IFN-controlled in humans. *DOPEY2*, *TMEM50B*, *PCBP3, RCAN1*, and *SIM2*, all of which are considered candidate genes for neuropathology in DS/T21, are predicted by this analysis to be IFN-regulated.

### 3.1. DOPEY2

**Figure 1:**
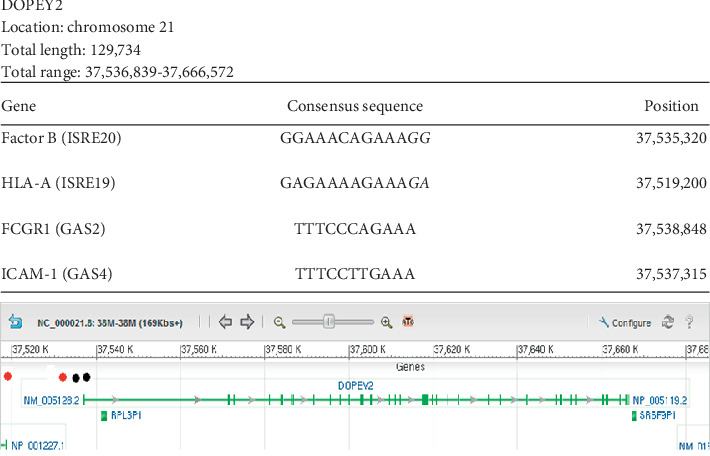
Localization of Ch21 ISRE and GAS consensus sequences in *DOPEY2*. Graphical representation of the results of a GenBank analysis of Ch21 identifying *DOPEY2* as a gene containing at least two interferon-stimulated response element (ISRE) consensus sequences and at least one interferon-gamma activation (GAS) sequence. All the ISRE/GAS sites outside the gene were found very close upstream of the gene, the most likely loci for gene control sites. The table above the graph shows that specific ISRE or GAS sequences found in the regulatory regions of other known genes are also present in *DOPEY2*. Column 1 presents the name of the gene and reference sequence found. Column 2 shows the detected contiguous sequence in bold font while the mismatched bases are depicted in italics font. Column 3 gives the position of the detected sequence in the gene. The first number in the total range represents the transcription start site. All the genes that our analysis predicts to be IFN-controlled are on chromosome 21 based on the experimental design. Red dots represent ISRE sequences. Black dots represent GAS sequences.

Figure 1 shows that *DOPEY2* possesses two ISRE and two GAS consensus sequences. We found four consensus sequences in close proximity to the transcription start site. The complete control site reported for the gene encoding for the protein known as high affinity immunoglobulin gamma Fc receptor-1 (*FCGR1*) is located inside the gene 2009 base pairs downstream from the transcription start site followed by the control site for the gene encoding for intercellular adhesion molecule-1 (*ICAM1*) separated by 1533 base pairs. The gene for complement factor B and human leukocyte antigen-A (*Factor B, HLA-AI*) control sites are located upstream to the transcription start site. Both ISRE sequences showed an 11-base contiguous homology to the original reported sequence with the conserved TTTC sequence.

### 3.2. TMEM50B

**Figure 2:**
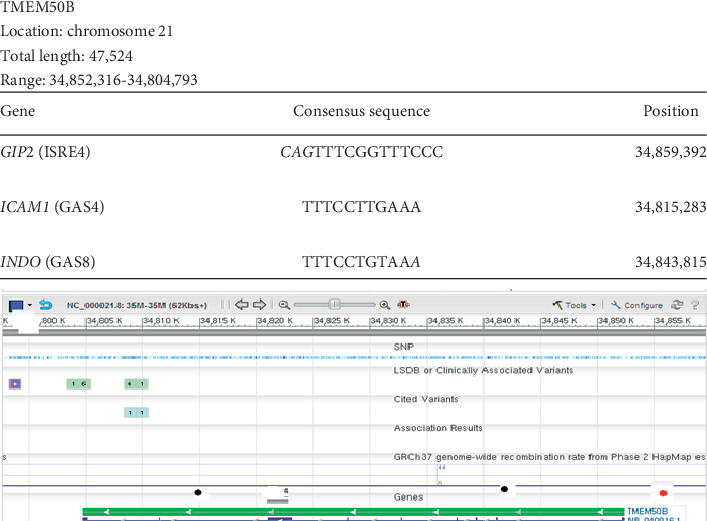
Localization of Ch21 ISRE and GAS consensus sequences in *TMEM50B*. Graphical representation of the results of a GenBank analysis of Ch21 identifying *TMEM50B* as a gene containing at least two interferon-stimulated response element (ISRE) consensus sequences and at least one interferon-gamma activation (GAS) sequence. All the ISRE/GAS sites outside the gene were found very close upstream of the gene, the most likely loci for gene control sites. The table above the graph shows that specific ISRE or GAS sequences found in the regulatory regions of other known genes are also present in *TMEM50B*. Column 1 presents the name of the gene and reference sequence found. Column 2 shows the detected contiguous sequence in bold font while the mismatched bases are depicted in italics font. Column 3 gives the position of the detected sequence in the gene. The first number in the total range represents the transcription start site. All the genes that our analysis predicts to be IFN-controlled are on chromosome 21 based on the experimental design. Red dots represent ISRE sequences. Black dots represent GAS sequences.

Figure 2 shows that *TMEM50B* has one ISRE and two GAS sequences. We found two GAS sequences inside the gene. One of the GAS sequence represents the control site for *ICAM1* and is a complete sequence. The other GAS sequence represents the control site for the indole 2, 3 oxygenase gene *INDO*, a 10-base pair contiguous homology sequence. Of the two ISREs, one is a control site for *GIP2*, the gene encoding a protein gamma-tubulin complex recruitment to the microtubule organizing centers of mitotic spindles, located approximately 6000 base pairs upstream, and is a 12-base pair contiguous homology sequence. The other ISRE sequence of interest (not plotted in the figure) is the one located inside the gene. It represents the control site for *IP10*. It is nearly a complete sequence with one mismatched base at position two.

### 3.3. PCBP3

**Figure 3:**
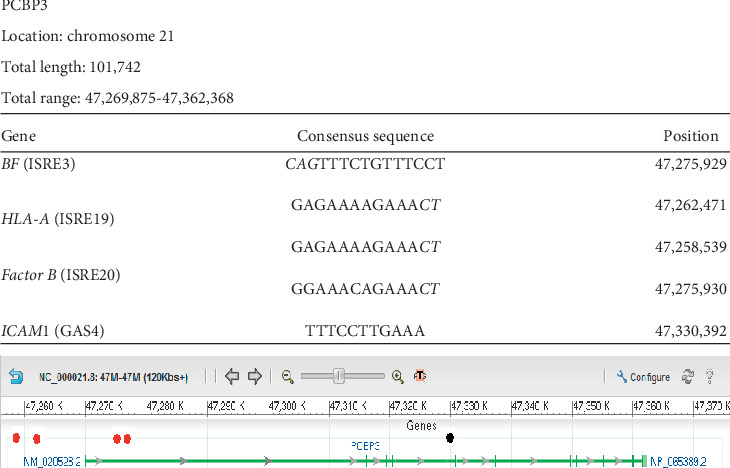
Localization of Ch21 ISRE and GAS consensus sequences in *PCBP3*. Graphical representation of the results of a GenBank analysis of Ch21 identifying *PCBP3* as a gene containing at least two interferon-stimulated response element (ISRE) consensus sequences and at least one interferon-gamma activation (GAS) sequence. All the ISRE/GAS sites outside the gene were found very close upstream of the gene, the most likely loci for gene control sites. The table above the graph shows that specific ISRE or GAS sequences found in the regulatory regions of other known genes are also present in *PCBP3*. Column 1 presents the name of the gene and reference sequence found. Column 2 shows the detected contiguous sequence in bold font while the mismatched bases are depicted in italics font. Column 3 gives the position of the detected sequence in the gene. The first number in the total range represents the transcription start site. All the genes that our analysis predicts to be IFN-controlled are on chromosome 21 based on the experimental design. Red dots represent ISRE sequences. Black dots represent GAS sequences.

Figure 3 shows that *PCBP3* contains three ISRE sequences and one GAS sequence. We found the complete GAS sequence that represents the control site reported for *ICAM1* located inside the gene; two of the three ISRE sequences that represent the major histocompatibility complex (MHC) *BF* and *Factor B* control sites were located 6054 base pairs away from the start site and found in tandem. While the sequence that represents *BF* showed 12-base contiguous homology to its original sequence, the sequence that represents *Factor B* shows an 11-base contiguous homology to its original sequence. One other ISRE sequence that represents the control sites for *HLA-A* is located 11,336 base pairs upstream to the start site and is an 11-base contiguous homology sequence.

### 3.4. RCAN1

**Figure 4:**
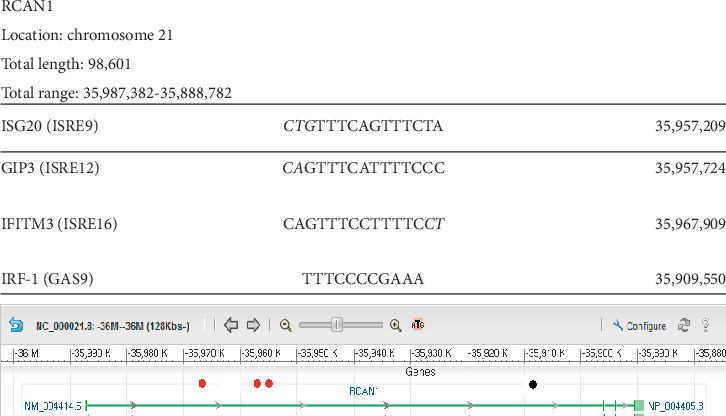
Localization of Ch21 ISRE and GAS consensus sequences in *RCAN1*. Graphical representation of the results of a GenBank analysis of Ch21 identifying *RCAN1* as a gene containing at least two interferon-stimulated response element (ISRE) consensus sequences and at least one interferon-gamma activation (GAS) sequence. All the ISRE/GAS sites outside the gene were found very close upstream of the gene, the most likely loci for gene control sites. The table above the graph shows that specific ISRE or GAS sequences found in the regulatory regions of other known genes are also present in *RCAN*. Column 1 presents the name of the gene and reference sequence found. Column 2 shows the detected contiguous sequence in bold font while the mismatched bases are depicted in italics font. Column 3 gives the position of the detected sequence in the gene. The first number in the total range represents the transcription start site. All the genes that our analysis predicts to be IFN-controlled are on chromosome 21 based on the experimental design. Red dots represent ISRE sequences. Black dots represent GAS sequences.

Figure 4 shows that *RCAN1* has three ISRE and one GAS sequence, all of which are located inside the gene. We detected the interferon regulatory factor-1 gene (*IRF1*) control site within the gene in a complete sequence. The control sites reported for interferon-stimulated exonuclease gene-20 (*ISG20*) and *GIP3* are 12-base contiguous homology sequences. The other sequence detected is a control site for interferon-induced transmembrane protein-3 (*IFITM3*) and is a 13-base contiguous homology sequence.

### 3.5. SIM2

**Figure 5:**
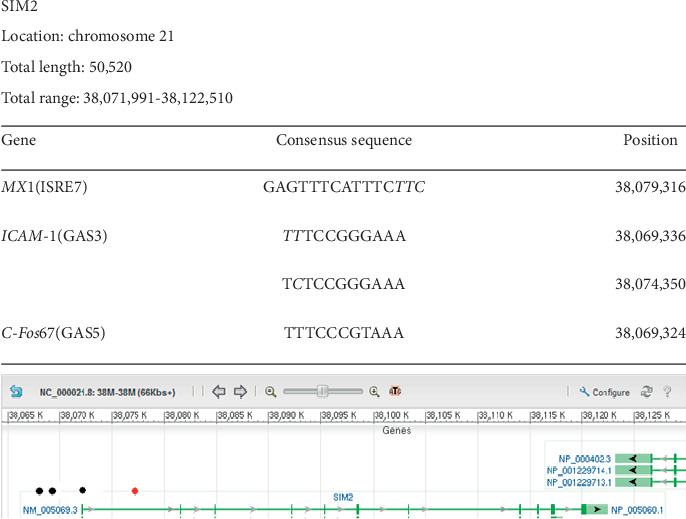
Localization of Ch21 ISRE and GAS consensus sequences in *SIM2*. Graphical representation of the results of a GenBank analysis of Ch21 identifying *SIM2* as a gene containing at least two interferon-stimulated response element (ISRE) consensus sequences and at least one interferon-gamma activation (GAS) sequence. All the ISRE/GAS sites outside the gene were found very close upstream of the gene, the most likely loci for gene control sites. The table above the graph shows that specific ISRE or GAS sequences found in the regulatory regions of other known genes are also present in *SIM2*. Column 1 presents the name of the gene and reference sequence found. Column 2 shows the detected contiguous sequence in bold font while the mismatched bases are depicted in italics font. Column 3 gives the position of the detected sequence in the gene. The first number in the total range represents the transcription start site. All the genes that our analysis predicts to be IFN-controlled are on chromosome 21 based on the experimental design. Red dots represent ISRE sequences. Black dots represent GAS sequences.

Figure 5 shows that *SIM2* possesses one ISRE sequence and three GAS sequences; one complete GAS sequence which is a control site for the protooncogene encoding c-fos neuronal synaptic activation protein (*C-Fos*) was located 2,667 base pairs upstream to the transcription start site. There exists another nine-base pair sequence in tandem representing the control site for *ICAM1* separated by only one base pair. An exception to our criteria was made in the case of *SIM2* to consider a nine-base sequence as being significant because of the tandem nature of its occurrence. Another GAS sequence that represents *ICAM1* again is a 9 + 1 sequence, whereby the contiguous homology extends with the first 9 bases followed by a mismatched base and an additional base that matches the original sequence. This again was considered significant because of the clustering with other GAS sequences noted in the vicinity of the transcription start site. We also noted the presence of an ISRE sequence which is the control site for the *MX1* gene encoding an interferon-induced dynamin-like GTPase with antiviral activity against a wide range of RNA, found inside the *SIM2* gene, which is a 12-base pair contiguous homology sequence.

## 4. Discussion

The main findings of this study are that *DOPEY2*, *TMEM50B*, *PCBP3, RCAN1*, and *SIM2* contain at least two ISRE sequences and one GAS control site upstream of their transcription start sites on Ch21, which qualifies them as IFN-regulated genes. The genes identified in this *in silico* analysis are potentially induced by type1 and/or type2 IFNs; however, future experimental studies will be needed to confirm this prediction.

Nevertheless, these findings suggest novel hypotheses concerning mechanisms for the IFN hypersensitivity and predilections for autoimmune and neurodegenerative diseases in persons affected by DS/T21.

### 4.1. DS/T21-Related Neurodegeneration

#### 4.1.1. IFN-DOPEY2 and TMEM50B Interactions

*DOPEY2* is a member of the family of *DOP* developmental genes first described by Axelrod and associates [18] and named for their role in development of the fungus *Aspergillus* (not named pejoratively for the mental and intellectual impairment associated with DS/T21). *DOPEY2* is a DS critical region gene on the distal end of Ch21 that is overexpressed in the brains of the DS/T21 experimental mouse model known as Ts1Cje [19], as well as in the brains of DS/T21 fetuses [20]. The Ts1Cje mouse contains a segmental trisomy wherein the distal end of Ch16 in the mouse is genetically equivalent to that of human Ch21 [19]. The function of *DOPEY2* is largely unknown, but it is thought to encode a transcription factor involved in subcellular organization of endoplasmic reticulum and Golgi apparatus [19] with increased density of cells in the cerebral cortex in the Ts1Cje mouse wherein DOPEY2 is overexpressed. Our finding that *DOPEY2* contains at least two ISRE sequences and one GAS upstream of the *DOPEY2* transcription start site suggests a role for IFN hypersensitivity, dysregulation, and autoimmunity in DS/T21-related mental retardation. *TMEM50B* is another gene shown to be overexpressed in the Ts1Cje mouse model encoding a “transmembrane protein” associated with endoplasmic reticulum and Golgi apparatus [19] that also met our criteria for IFN-regulated gene on human Ch21. These findings raise the question as to whether IFN dysregulations of *DOPEY2* and *TMEM50B* and/or autoimmune responses play roles in other diseases of intellectual disability associated with neurodegenerative diseases. These results also suggest that it is time to rethink whether the autoantibodies directed against a variety of molecules in patients diagnosed with AD are, rather than inflammatory biomarkers, causally related to the risk for, and development of, AD [21].

#### 4.1.2. IFN-PCB3-Tau Protein Interactions

*PCB3* is a gene that also meets our criteria of possessing at least two ISRE sequences and one GAS upstream of its transcription start site. *PCB3* encodes for the poly-(rC)-binding protein3 that binds to cytosine-rich pyrimidine regions on RNAs and is shown to be necessary for normal tau protein splicing [22]. IFN-induced phosphorylation of tau protein is shown to be a mechanism for the neurodegeneration associated with AD [23] and possibly the DS/T21-related predilection for AD. IFN-driven immune dysregulation is thought to be a key contributor to the DS/T21-related predilection for autoimmune diseases. IFN-*α* hypersensitivity is a feature of DS/T21, associated with upregulation of the *IFNAR1* gene for the *α*-chain component of IFN-*α*/*β* receptors on Ch21 and increased expression of IFN-*α* receptors on the surface of T-cells, monocytes, and other types of immunocytes [23]. This raises the question as to whether IFN hypersensitivity with an increased expression of IFN receptors on immunocytes capable of inducing phosphorylation and/or abnormal splicing of tau protein is a factor in cases of AD and other neurodegenerative diseases in the absence of DS/T21, thereby qualifying it as an autoimmune disease.

#### 4.1.3. IFN-GSK3 Interactions

Glycogen synthetase kinase (GSK3) is known to stimulate production of IFN-*γ* by Th1 cells and increase STAT signaling. JAK/STAT is the same downstream signaling pathway used by type1 IFNs such as IFN-*α*, the activity of which is increased in autoimmune diseases [24]. It is noteworthy that lithium is shown to be effective in ameliorating experimental autoimmune encephalomyelitis by the mechanism of inhibiting GSK3 [24], and GSK3 inhibition may therefore explain the lithium-induced improvement in neural plasticity and memory demonstrated in DS mice [25].

#### 4.1.4. IFN-RCAN1 and Calcineurin-Kynurenic Acid Interactions

Our finding that *RCAN1* contains at least two ISRE sequences and one GAS upstream of its transcription start site suggests that it is an IFN-regulated gene. RCAN1 is shown to be overexpressed in DS/T21 fetuses [26]. RCAN1 is found in the DSCR of Ch21. *RCAN1* is named for the fact that its protein product calcipressin functions as an inhibitor of calcineurin signaling [27]. Calcipressin also modulates nuclear factor activated T-cell (NFAT) signaling, a regulator of T-cell activation [28]. T-cell activation is known to be a factor in neurotoxicity associated with exposure to a wide variety of toxicants [29]. Chronic overexpression of RCAN1 in adults appears to promote the development of hyperphosphorylated tau proteins, likely promoted by T-cell dysregulation, which results in the neurofibrillary tangles characteristic of AD [26]. Such neurofibrillary tangles are also found in the brains of patients diagnosed with Huntington's disease and other neurodegenerative diseases [30].

Persons with DS/T21 also overexpress an IFN-stimulated gene on Ch8 for producing indoleamine-pyrrole 2,3 dioxygenase 1 (IDO-1). IDO is the rate-limiting enzyme in the kynurenine pathway. Overexpression of IDO with elevated plasma levels of the tryptophan metabolites, kynurenine, and quinolinic acid, which are indole derivatives possessing robust antimicrobial activity, thereby serving to be protective against infectious agents. Dysregulation of the kynurenine pathway metabolites is reported to produce free radicals and reactive oxygen species (ROS), which are known to promote inflammation and neurodegeneration [31]. The question as to whether the kynurenine pathway is dysregulated in other neurodegenerative diseases in the absence of DS/T21 is partly answered by studies demonstrating dysregulation of NFAT signaling and T-cell activation from administration of lipopolysaccharide (LPS) to mice [32]. LPS administration is associated with elevations in kynurenic acid in the prefrontal cortex and dysregulation of NMDA signaling with deficits in stimulus processing during classical Pavlovian behavioral conditioning [32], likely due to NMDA receptor antagonism by the kynurenic acid, an endogenous NMDA receptor inhibitor also shown to induce a robust cortical inflammatory response, which may disrupt cortical development [33]. Taken together, these findings suggest that calcineurin-kynurenine interaction may contribute to the neurodegeneration and predilection for AD in persons affected by DS/T21.

### 4.2. DS/T21-Related Diabetes Mellitus and Thyroid Disease

Autoimmune diabetes mellitus (DM1) and the thyroid conditions Hashimoto's thyroiditis and Graves' disease are reported to be the autoimmune conditions most commonly found in persons diagnosed with DS/T21 [34]. A role for IFN receptors in autoimmune DM1 is demonstrated in mice wherein knockout of the receptors for IFN-alpha and IFN-gamma decreased the incidence of DM1 in females but increased it in males [35]. These findings suggest that IFNs may function as both positive and negative modulators of DM1 risk [35]. It is noteworthy that the autoimmune disease Hashimoto's thyroiditis is reported to present as more severe in females with DS/T21 than in females without this chromosomopathy [36].

### 4.3. DS/T21-Related Hypertrophic Cardiomyopathy and Hepatic Fibrosis IFN-RCAN1 and Calcineurin-Kynurenic Acid Interactions

The DS/T21 phenotype is commonly recognized by characteristic facial features and mild-to-moderate intellectual disability. However, evidence is emerging from continuing research that DS/T21 involves pathological changes in multiple body systems. The aforementioned IFN-*RCAN1* mediated inhibition of calcineurin signaling and adverse calcineurin-kynurenine interactions may play a role in the hypertrophic cardiomyopathy and hepatic fibrosis observed in DS/T21 persons. The RCAN1 protein calcipressin, a calcineurin inhibitor, is shown to be a requirement for extracellular matrix synthesis in cardiac hypertrophy and hepatic fibrosis [37], two conditions occurring in DS/T21-related hypertrophic cardiomyopathy and autoimmune hepatitis.

### 4.4. DS/T21-Related Joint, Renal, and Gastrointestinal Disorders

#### 4.4.1. IFN Hypersensitivity, Arthropathy, and Glomerulopathy

DS/T21-related IFN hypersensitivity seems to be specific for IFN-*α*, consistent with the predilections for inflammatory hyperresponsiveness, infections, and autoimmune diseases in persons affected by DS/T21 [38]. The type1 IFN system is our main defense against viruses, and there are numerous IFN-regulated genes known to upregulate the expression of at least fifteen different proteins with immunologic antiviral activity. IFN-alpha is a type1 IFN, and there appears to be an increase in the expression of type1 IFN-regulated genes (IFN signature) in systemic lupus erythematosus (SLE), an autoimmune rheumatic disease [38]. ISRE-like sequences have also been identified in human isoleucyl-tRNA synthetase, one of several acyl-tRNA synthetases which link amino acids together for translating amino acids into peptides and proteins, specifically those involved in development of immunocytes such as T-cells. The finding of ISRE-like sequences on human isoleucyl-tRNA synthetase suggests that IFNs could have the potential to control the development and polarity of T-cells and various other immunocytes, thereby creating predilections for modulating a wide variety of immune responses including autoimmune diseases [39]. Similar to T-cell dysregulation, there appears to be a mechanism for the IFN hyperactivity associated with DS/T21. For example, CD8+ T-cells are found to be more polarized than normal toward a Th1 response and production of cytokines such as IFN-gamma and TNF-alpha associated with senescence and autoimmunity, even in the absence of diagnosis of an autoimmune condition [1]. These findings suggest that the arthropathy and glomerulopathy [40] associated with Down syndrome may be underdiagnosed manifestations of the DS/T21-related autoimmunity associated with IFN hypersensitivity.

## 5. Summary and Conclusion

The present study identified genes on Ch21 possessing at least two ISRE sequences and one GAS control site within and/or upstream of the gene's transcription start site. A literature search revealed, among the genes that met the aforementioned criterion, the most likely IFN-regulated genes for explaining the neuropathology associated with IFN hypersensitivity in DS/T21. *DOPEY2*, *TMEM50B*, *PCBP3, RCAN1*, and *SIM2* were found to meet the aforementioned gene search and functional criteria. These *in silico* findings indicate that *DOPEY2*, *TMEM50B*, *PCBP3, RCAN1*, and *SIM2* are genes which may be dysregulated by interactions with type1 and/or type2 IFNs in DS/T21. Our findings suggest that these genes may serve as novel targets for treatments aimed at ameliorating the neuropathological features of DS/T21. These results also imply that IFN dysregulation and autoimmunity may be a causal contributing factor to neurodegenerative, endocrine, renal, and gastrointestinal conditions which may share common autoimmune etiologies with DS/T21. This study shows how probing the human genome can create new knowledge about the mechanisms of autoimmunity and etiologies of complex multisystemic diseases.

## Figures and Tables

**Table 1:** Candidate down syndrome neuropathology genes on Ch21.

Gene	Function
*RCAN1*	Regulator of calcineurin 1
*MAFD3*	Major affective disorder 3
*JAM4*	Junctional adhesion molecule 4
*ADARB1*	Adenosine deaminase, RNA specific, B1
*DOPEY2*	Dopey family member 2
*mir155*	micro-RNA 155
*NCAM*	Nuclear receptor interacting protein
*DYRK*	Dual specificity tyrosine-(y)-phosphorylation regulated kinase 1A
*MX1*	Myxovirus (influenza) resistance-1
*SIM2*	Single-minded homolog2 (drosophila)
*SYNJ1*	Synaptojanin 1
*DIP2a*	Disco interacting protein
*PCP4*	Purkinje cell protein 4
*CD18*	Integrein beta 2
*SOD1* and *SOD2*	Superoxide dismutase

**Table 2:** Genes with ≥2 ISRE and/or GAS consensus sequences on Ch21.

Gene	No. of consensus sequences
*DSCAM*	6
*C21orf34*	6
*NCAM 2*	5
*PCBP3*	5
*DOPEY2*	4
*RCAN1*	4
*SIM2*	4
*ERG*	3
*NRIP1*	3
*TMEM50B*	3
*Coll8A1*	2
*GIRK1*	2
*C21orf109*	2
*ITSN1*	2
*BACE2*	2
*PRSS1*	2

## Data Availability

The data on which this research is based are available through contacting Dr. Jagadeesh at ajagadeesh@auamed.net.

## References

[1] Araya P., Waugh K. A., Sullivan K. D. (2019). Trisomy 21 dysregulates T cell lineages toward an autoimmunity-prone state associated with interferon hyperactivity. *Proceedings of the National Academy of Sciences*.

[2] Li Q. Z., Zhou J., Lian Y. (2010). Interferon signature gene expression is correlated with autoantibody profiles in patients with incomplete lupus syndromes. *Clinical & Experimental Immunology*.

[3] Lee A. J., Ashkar A. A. (2018). The dual nature of type I and type II interferons. *Frontiers in Immunology*.

[4] Pestka S., Krause C. D., Walter M. R. (2004). Interferons, interferon-like cytokines, and their receptors. *Immunological Reviews*.

[5] López de Padilla C. M., Niewold T. B. (2016). The type I interferons: basic concepts and clinical relevance in immune-mediated inflammatory diseases. *Gene*.

[6] Gibbert K., Schlaak J., Yang D., Dittmer U. (2013). IFN-*α* subtypes: distinct biological activities in anti-viral therapy. *British Journal of Pharmacology*.

[7] Singh A. K., Novakova L., Axelsson M. (2017). High interferon-*γ* uniquely in v*δ*1 T cells correlates with markers of inflammation and axonal damage in early multiple sclerosis. *Frontiers in Immunology*.

[8] Yockey L. J., Iwasaki A. (2018). Interferons and proinflammatory cytokines in pregnancy and fetal development. *Immunity*.

[9] Yao Y., Liu Z., Jallal B., Shen N., Rönnblom L. (2013). Type I interferons in Sjögren’s syndrome. *Autoimmunity Reviews*.

[10] Crow M. K. (2014). Advances in understanding the role of type I interferons in systemic lupus erythematosus. *Current Opinion in Rheumatology*.

[11] Rönnblom L., Leonard D. (2019). Interferon pathway in SLE: one key to unlocking the mystery of the disease. *Lupus Science & Medicine*.

[12] Stanifer M. L., Pervolaraki K., Boulant S. (2019). Differential regulation of type I and type III interferon signaling. *International Journal of Molecular Sciences*.

[13] Williams B. R. G. (1991). Transcriptional regulation of interferon-stimulated genes. *European Journal of Biochemistry*.

[14] Michalska A., Blaszczyk K., Wesoly J., Bluyssen H. A. R. (2018). A positive feedback amplifier circuit that regulates interferon (IFN)-stimulated gene expression and controls type I and type II IFN responses. *Frontiers in Immunology*.

[15] Gobin S. J., Van Zutphen M., Woltman A. M., Van Den Elsen P. J. (1999). Transactivation of classical and nonclassical HLA class I genes through the IFN-stimulated response element. *Journal of Immunology*.

[16] Tsukahara T., Kim S., Taylor M. W. (2006). Refinement: a search framework for the identification of interferon-responsive elements in DNA sequences—a case study with ISRE and GAS. *Computational Biology and Chemistry*.

[17] Langer J. A., Rashidbaigi A., Lai L.-W., Patterson D., Jones C. (1990). Sublocalization on chromosome 21 of human interferon-alpha receptor gene and the gene for an interferon-gamma response protein. *Somatic Cell and Molecular Genetics*.

[18] Axelrod D. E., Gealt M., Pastushok M. (1973). Gene control of developmental competence in *Aspergillus nidulans*. *Developmental Biology*.

[19] Ling K.-H., Hewitt C. A., Tan K.-L. (2014). Functional transcriptome analysis of the postnatal brain of the Ts1Cje mouse model for down syndrome reveals global disruption of interferon-related molecular networks. *BMC Genomics*.

[20] Rachidi M., Delezoide A. L., Delabar J. M., Lopes C. (2009). A quantitative assessment of gene expression (QAGE) reveals differential overexpression of *DOPEY2,* a candidate gene for mental retardation, in down syndrome brain regions. *International Journal of Developmental Neuroscience*.

[21] Wu J., Li L. (2016). Autoantibodies in Alzheimer’s disease: potential biomarkers, pathogenic roles, and therapeutic implications. *Journal of Biomedical Research*.

[22] Wang Y., Gao L., Tse S.-W., Andreadis A. (2010). Heterogeneous nuclear ribonucleoprotein E3 modestly activates splicing of tau exon 10 via its proximal downstream intron, a hotspot for frontotemporal dementia mutations. *Gene*.

[23] Waugh K. A., Araya P., Pandey A. (2019). Mass cytometry reveals global immune remodeling with multi-lineage hypersensitivity to type I interferon in down syndrome. *Cell Reports*.

[24] Rowse A. L., Naves R., Cashman K. S. (2012). Lithium controls central nervous system autoimmunity through modulation of IFN-*γ* signaling. *PLoS One*.

[25] Contestabile A., Greco B., Ghezzi D., Tucci V., Benfenati F., Gasparini L. (2013). Lithium rescues synaptic plasticity and memory in down syndrome mice. *Journal of Clinical Investigation*.

[26] Martin K. R., Layton D., Seach N. (2019). Methylation of RCAN1.4 mediated by DNMT1 and DNMT3b enhances hepatic stellate cell activation and liver fibrogenesis through calcineurin/NFAT3 signaling. *Theranostics*.

[27] GenescàKilleen L., Aubareda A., Fuentes J. J., Estivill X., De La Luna S., Pérez-Riba M. (2003). Phosphorylation of calcipressin 1 increases its ability to inhibit calcineurin and decreases calcipressin half-life. *Biochemical Journal*.

[28] Hogan P. G. (2017). Calcium-NFAT transcriptional signalling in T cell activation and T cell exhaustion. *Cell Calcium*.

[29] Pollard K. M., Hultman P., Kono D. H. (2010). Toxicology of autoimmune diseases. *Chemical Research in Toxicology*.

[30] Gratuze M., Cisbani G., Cicchetti F., Planel E. (2016). Is Huntington’s disease a tauopathy?. *Brain*.

[31] Powers R. K., Culp-Hill R., Ludwig M. P. (2013). Upregulation of RCAN1 causes down syndrome-like immune dysfunction. *Journal of Medical Genetics*.

[32] Oliveros A., Wininger K., Sens J. (2017). LPS-induced cortical kynurenic acid and neurogranin-NFAT signaling is associated with deficits in stimulus processing during pavlovian conditioning. *Journal of Neuroimmunology*.

[33] Bagasrawala I., Zecevic N., Radonjić N. V. (2016). N-methyl D-aspartate receptor antagonist kynurenic acid affects human cortical development. *Frontiers in Neuroscience*.

[34] Guaraldi F., Rossetto Giaccherino R., Lanfranco F. (2017). Endocrine autoimmunity in down’s syndrome. *Endocrine Immunology*.

[35] Carrero J. A., Benshoff N. D., Nalley K., Unanue E. R. (2018). Type I and II interferon receptors differentially regulate type1 diabetes susceptibility in male versus female NOD mice. *Diabetes*.

[36] Aversa T., Lombardo F., Valenzise M., Luca F., Wasniewska M. (2015). Peculiarities of autoimmune thyroid diseases in children with turner or down syndrome: an overview. *Italian Journal of Pediatrics*.

[37] Pan X.-Y., You H.-M., Wang L. (2019). Methylation of RCAN1.4 mediated by DNMT1 and DNMT3b enhances hepatic stellate cell activation and liver fibrogenesis through calcineurin/NFAT3 signaling. *Theranostics*.

[38] Rönnblom L. (2016). The importance of the type1 interferon system in autoimmunity. *Clinical and Experimental Rheumatology*.

[39] Nichols R. C., Raben N., Boerkoel C. F., Plotz P. H. (1995). Human isoleucyl-tRNA synthetase: sequence of the cDNA, alternative mRNA splicing, and the characteristics of an unusually long c-terminal extension. *Gene*.

[40] Málaga S., Pardo R., Málaga I., Orejas G., Fernández-Toral J. (2005). Renal involvement in down syndrome. *Pediatric Nephrology*.

